# The Tool, the Pet, the Carer & the Warden: A Conceptual Framework of How Older Adults Perceive Socially Assistive Robots

**DOI:** 10.1007/s10796-026-10699-2

**Published:** 2026-02-27

**Authors:** Laura Pemberton, Nikolay Mehandjiev, Michelle Carter

**Affiliations:** 1 Alliance Manchester Business School, The University of Manchester, Oxford Road, Manchester, M13 9PL UK; 2 Present Address: School of Business, University of Lancashire, Fylde Road, PR1 2HE Lancashire, UK; 3 The University of Birmingham Dubai Campus, Dubai International Academic City, Dubai, United Arab Emirates

**Keywords:** Anthropomorphism, Socially assistive robots (SARs), Older adults, Aging-in-place, Computers are social actors (CASA)

## Abstract

As the population of older adults in the UK grows, Socially Assistive Robots (SARs) are increasingly important for supporting independence and enhancing quality of life within the home. This study draws on the Computers Are Social Actors (CASA) framework to explore how older adults perceive and interact with anthropomorphized SARs. We present a framework that categorizes these devices into four roles: Carer, Pet, Warden, and Tool. The framework reflects relationship tolerance and autonomy, capturing the varied relational and emotional interactions older users have with SARs. Using qualitative methods, including interviews with 13 older adults in a virtual reality smart home setup, the findings reveal diverse user expectations. Devices like Alexa and a robotic cat align with the Pet role, providing companionship and emotional comfort, while humanoid robots fit the Warden role, offering oversight without attachment. Design implications emphasize customizable anthropomorphic features, enabling devices to adapt to users’ emotional and practical needs, ultimately fostering autonomy and quality of life for aging individuals.

## Introduction

The UK’s aging population is set for a dramatic expansion, with those aged 65 or over surging from 9.2 million in 2011 to 12.7 million in 2022 and projected to reach 22.1 million by 2072 (Barton et al., 2024). Despite this demographic shift, the proportion of older adults residing in care homes has seen a decline from 3.2% to 2.5% over recent years (Storey, 2022). This trend highlights a growing societal imperative: to empower older individuals to maintain independence and enhance their quality of life within their own homes. Socially assistive robots (SARs) offer a promising technological avenue to address this increasing demand for in-home care. Indeed, SARs can provide a viable support system for older adults living independently, and in some cases, may even be preferred over human caregivers (Smarr et al., 2014; Van Assche et al., 2024). However, as technological capabilities continue to accelerate, many older adults report increasing difficulty in keeping pace, leading to concerns about usability, digital exclusion, and long term adoption (Choudrie & Vyas, 2014).

SARs promise personalized, non-intrusive care. However, their acceptance and effectiveness fundamentally depend on how older adults perceive and relate to these devices. Existing Information Systems (IS) literature, however, adopts a narrow, utilitarian, and usability driven perspective on these perceptions, frequently failing to capture the complex, evolving human technology relationships that emerge (Bingley et al., 2023; Schuetz & Venkatesh, 2020). This contradicts recent IS calls to move beyond instrumental views to better account for emotional and identity related factors, particularly in domestic and caregiving contexts (Benlian et al., 2020).

Although roboticists explore user-centered design (Bradwell et al., 2019) and Human-Robot Interaction (HRI) research examines affective dimensions interaction (Whelan et al., 2018), IS scholarship notably lacks theory on how older adults construct meaning around diverse SAR forms (e.g., humanlike versus pet-like companions). Our study addresses this by employing Computers Are Social Actors (CASA) (Gambino et al., 2020) as a theoretical lens. CASA posits that individuals instinctively apply human social rules to interactions with technology. While comparative work confirms form and perceived intent shape emotional responses (Coeckelbergh, 2011), we understand little about how these perceptions translate, through metaphorical sense-making, into the practical and relational roles robots assume in older adults’ lives (Techatassanasoontorn et al., 2023).

An important explanatory shortfall remains in understanding how older adults balance maintaining personal autonomy with tolerating the evolving presence of SARs within their intimate home environments, especially when those robots are designed for close interaction (Benlian et al., 2020). Prior studies have not sufficiently explored older adults’ “relational tolerance” nor its intersection with their desire for autonomy as SAR capabilities and perceived roles become more pronounced. Autonomy and relational tolerance matter for ageing in place, as successful long-term adoption depends on technologies that both preserve self-determination and fit comfortably within older adults’ preferred relational boundaries (Peek et al., 2016). These boundaries are directly shaped by how older adults interpret the social cues described by CASA (Nass & Moon, 2000); cues that reliably trigger social expectations, comfort or discomfort, and boundary setting in human–technology interaction (Bartneck et al., 2009; Gambino et al., 2020; Nass & Moon, 2000). Our conceptual framework thus addresses this explanatory shortfall by capturing how relationship tolerance and perceived autonomy shape participants’ categorization of SARs into distinct roles: Carer, Pet, Warden, and Tool. In doing so, it extends IS theory by conceptualizing SARs as relational actors whose perceived roles shape older adults’ autonomy and relational boundaries in the home.

This paper addresses the following research question: *How do older adults perceive and interact with anthropomorphized socially assistive robots (SARs)*,* and how do these perceptions inform the roles that these devices play in supporting autonomy and emotional wellbeing in the home environment*? By answering this question, the study contributes both theoretical and applied insights to IS scholarship and to the design of SARs. The findings provide practical design guidance by identifying how different forms of SARs affect user trust, engagement, and comfort levels. By distinguishing the roles SARs play, this study informs customization strategies that can better align SAR behaviors with user preferences. These insights contribute to improving SAR adoption by ensuring that design choices support autonomy and emotional wellbeing.

The remainder of this paper is structured as follows: Sect. 2 reviews literature on socially assistive robots, aging-in-place, and anthropomorphism in human technology interaction. Section 3 describes the methodology and immersive virtual environment used for data collection. Section 4 presents the findings, highlighting four distinct relational roles, Carer, Pet, Warden, and Tool, that older adults ascribed SARs, shaped by perceptions of autonomy and relationship tolerance. Section 5 discusses the implications of these roles for SAR design and long-term adoption, offering a conceptual framework to inform future development. Section 6 concludes with reflections on the study’s contributions to theory and practice, and directions for future research.

## Related Work

This section explores how older adults use metaphorical sensemaking to interpret and assign roles to SARs influencing their acceptance. It then highlights how anthropomorphism, user autonomy, dignity, and alignment with personal preferences shape effective SAR designs for aging-in-place.

To understand how older adults perceive and interact with SARs, this study examines the process of metaphorical sensemaking (Hekkala et al., 2018), where individuals draw on familiar concepts to interpret emergent digital technologies. Studies demonstrate that stakeholders rely on metaphors like ‘person,’ ‘robot,’ and ‘tool’ to make sense of new digital agents, influencing their understanding of capabilities, limitations, and implications for human roles and practices (Techatassanasoontorn et al., 2023). Social robots, therefore, offer more than task oriented support; they can foster meaningful relationships with older adults in the home (De Carolis et al., 2017). Pet-like devices like the iCat (Heerink et al., 2006) illustrate how pet metaphors facilitate more effective emotional connections between older users and robots (Norouzi et al., 2019).

Our study extends metaphorical sense-making by exploring anthropomorphism of SARs through the CASA framework (Nass et al., 1994). CASA posits that people instinctively apply human-human social rules and behaviors to their interactions with computers, even though they recognize these devices as non-human. Minimal social cues, such as a human like voice or responsiveness, can elicit social perceptions and behaviors, making this cognitive contradiction central to understanding human SAR relationships (Gambino et al., 2020). However, while CASA provides foundational insights, its original formulation addressed less complex technologies (Heyselaar, 2023). Rapid developments in technology and shifts in human interactions over recent decades require theoretical extensions to address modern, sophisticated, and varied media agents such as SARs. Nguyen et al. (2022) argue that social responses to conversational agents no longer mirror human to human interaction scripts but reflect dynamics shaped by user effort, goal orientation, and social presence. Their typology of human chatbot interaction styles illustrates that individuals actively adapt and develop new social heuristics depending on the agent’s role, responsiveness, and communicative context. This reinforces the need to move beyond simplistic anthropomorphic assumptions, recognizing instead the evolving interpretive processes people engage in with socially interactive technologies.

Despite the promise of SARs in supporting emotional wellbeing, there remains a notable disconnect between user preferences and current design priorities. Older adults and roboticists often diverge in their visions for robot design; while roboticists often lean toward human like, advanced features, older adults prefer pet-like designs that are discreet, practical, and easy to use, emphasizing function over technological novelty (Bradwell et al., 2019). Similarly, perceived social presence is a key determinant of acceptance, particularly in influencing positive attitudes and willingness to engage with the robot (Heerink et al., 2008). Yet, there is limited research exploring embodied devices along the continuum from completely disembodied to fully humanoid forms.

Beyond functionality and emotional engagement, studies highlight ethical tensions around autonomy and dignity. Many older adults prefer robots that take on roles such as “tool” or “pet” rather than “friend” or “assistant,” underscoring the importance of maintaining their dignity and autonomy (Coghlan et al., 2021). This aligns with findings showing that concerns over deception, dignity, and the erosion of human contact often drive rejection of SARs, even when these robots are considered helpful (Vandemeulebroucke et al., 2020). Participants prefer robots whose appearance and behavior match their expectations of utility, avoiding a human like presence that threatens their independence (Pino et al., 2015).

Older adults have expressed openness to robotic assistance but consistently raise concerns about intrusive monitoring features, overly humanlike design, and threats to privacy (Zsiga et al., 2013). They particularly emphasize a desire to retain autonomy, identifying overreach in control and cognitive support as key barriers to acceptance. Some adults highlight the importance of physical assistance while maintaining personal control over robot usage. This divergence exposes a risk: SARs may undermine, rather than enable, autonomy when design choices prioritize care needs over user preferences. Other studies echo this caution. Older adults in domestic trials endorsed self-efficacy and usefulness over sociability, viewing many social features with ambivalence (De Graaf et al., 2019). Researchers have found that while social presence and enjoyment can enhance acceptance, these effects quickly diminish if robots appear too adaptive, unpredictably sociable, or threatening to user control (Heerink et al., 2010). Robot appearance also plays a critical role: some older adults favor humanoid faces for social interaction, but many prefer more mechanical looking robots that preserve emotional distance, particularly in private contexts like bathing (Prakash & Rogers, 2015). These preferences highlight expectations that a robot’s form and function should align with the social norms of the task and the user’s role. Together, these findings demonstrate that older adults actively evaluate whether a robot’s behavior and embodiment respect their autonomy, boundaries, and everyday values. Designing for successful aging, therefore, requires technologies that not only align with functional needs but also with users’ expectations of relational and moral appropriateness.

Epley et al. (2007, p. 864) define anthropomorphism as the human tendency “to imbue the real or imagined behavior of nonhuman agents with humanlike characteristics, motivations, intentions, or emotions”. Research on anthropomorphized chat bots has largely focused, and holds promising results for fostering trust and emotional engagement (Benlian et al., 2020; Mourey et al., 2017; Touré-Tillery & McGill, 2015). Anthropomorphism has long played a role in consumer product design, demonstrating that a device does not need to resemble a human for a user to anthropomorphize it: in fact, many technologies can be anthropomorphized (Zheng & Jarvenpaa, 2021). For example, Amason’s Alexa is often anthropomorphized by users who have gendered the device as female based on the name and the voice. This tendency arises as individuals seek familiarity in ambiguous situations, applying human characteristics to technology to feel more socially connected (Zheng & Jarvenpaa, 2021). In home-based technology research, focus has increasingly shifted toward SARs designed explicitly for social interaction and companionship. While extensive, this research typically focuses on older adults with cognitive impairments (e.g., dementia) or on technological evaluations of system performance, with comparatively limited attention given to everyday experiences, concerns, and expectations of cognitively intact older adults (Niemelä-Nyrhinen, 2007).

Several experimental and qualitative studies have explored how SARs are perceived by older users across emotional, practical, and ethical dimensions (see Table 1). Ihamäki and Heljakka (2021) found that commercial robotic pets like the Golden Pup robot elicited strong emotional responses, such as joy, empathy, and surprise, and fostered attachment and reminiscence among older adults aged 65–85. These interactions were marked by anthropomorphism and active engagement, suggesting the emotional potential of SARs. Similarly, Dosso et al. (2023) reported that both older adults and care partners appreciated robots’ emotional expressiveness, but also raised concerns regarding stigma and infantilization, particularly when robots were used in public. Participants valued robots that could be tailored in emotional tone and interactivity, emphasizing their preference for systems responsive to individual emotional landscapes.

**Table 1:** Summary of related studies

Study (Year)	Type of Technology	Age of Older Adult	Dimensions Reviewed	Key Findings
Ihamäki and Heljakka (2021)	Commercial robotic pet (Golden Pup robot dog)	65–85 years (*n* = 10)	Social and Emotional Experiences (including “Joy, Surprise, Empathy, Softness, Active, Touch, Neutral”), Relationship Building, Wellbeing, Attachment	Robot pets (Golden Pup) stimulated joy, empathy, and reminiscence, encouraging social interaction and attachment. Older adults actively engaged, even those initially fearful, highlighting emotional and wellbeing benefits.
Dosso et al. (2023)	Pet-like robot (MiRo), Tabletop robot (T-Top)	Older adults (*n* = 25) in their 50–80 s, Older adults with dementia (*n* = 2) aged 65–74, Care partners (*n* = 17) in their 30–70 s	Emotional Alignment (range and display of robot emotions, comfort in expressing emotions to robot), Perceptions of Stigma (around public use, negative stereotypes, appearance, infantilization), Design Features and Application Areas (communication, companionship, practical tasks, health support)	Older adults and caregivers valued emotional alignment and expression in robots but noted stigma as a barrier. Customizable emotional display and interactivity were preferred to support comfort and acceptance.
Schneiders et al. (2021)	Fictional Personal Assistants with varying physical embodiment (low, moderate, high anthropomorphism)	Mixed age range: validation study (*N* = 26, age 22–46, avg. 29.8); main study (*N* = 150, age 23–69, avg. 36.1)	Perceived Intelligence, Likeability, Overall, Goodness, Pragmatic Qualities (usefulness/usability), Hedonic Qualities (aesthetics/pleasure)	Higher anthropomorphism increased perceived likeability and intelligence but not overall appeal. The mid-level design performed worst, suggesting an uncanny valley effect. High expectations may lead to user disappointment if they are unmet.
Bradwell et al. (2019)	Companion robots (Joy for All: Golden Pup and Cat; other conceptual discussions)	Older adults (*N* = 33, living in supported living or own homes) and Roboticists (*N* = 9)	User preferences for robot design (physical appearance, behavior, interaction style, function), Differences between older adult and roboticist preferences, User-centered design importance	Older adults preferred subtle, pet-like, practical robots, while roboticists favored advanced human like designs. Findings stress the importance of aligning robot design with older adults’ needs, not just technical innovation.
Heerink et al. (2008)	Companion Robot (unspecified type, focus on acceptance for older adults)	Older adults (*N* = 50, age range 65–90, living independently or in care facilities)	Social Presence, Perceived Usefulness, Perceived Ease of Use, Attitude towards Robot, Intention to Use	Higher social presence significantly increased older adults’ acceptance and intention to use companion robots, highlighting its importance in fostering positive attitudes and perceived companionship.
Coghlan et al. (2021)	Robot Companions (various, conceptual discussion based on participant input)	Older adults (*N* = 20, age 65–90, living independently)	Dignity, Autonomy, Style of Company (types of companionship, e.g., friend, pet, assistant), Public Perception, Ethical Considerations (trust, privacy)	Older adults preferred robots as ‘tools’ or ‘pets’ to preserve dignity and autonomy. Stigma and fear of appearing dependent reduced willingness to adopt, underscoring the need for non-infantilizing designs.
Pino et al. (2015)	Socially Assistive Robots (SARs) - general	Older adults (*N* = 72, mean age 79.5 years) living at home, assisted living facilities, or nursing homes	Attitudes towards SARs (positive/negative aspects), Reasons for acceptance/rejection (helpfulness, interaction, human likeness, comfort, cost, perceived need, lack of interest), Ethical considerations (deception, dignity, autonomy)	Older adults showed mixed views on SARs, valuing usefulness and ease of use but concerned about dignity, autonomy, and deception. Acceptance varied with human likeness, depending on context and perceived utility.
Łukasik et al. (2021)	Socially Assistive Robot (SAR) – Kompai (conceptual image)	60 + years (*n* = 37 older adults; total *N* = 166 including younger adults aged 20–59)	Cognitive Training, Memory Support, Guided Physical Exercise, Mood Detection, Social Engagement, Companion Role, Gender and Age Differences	Older adults favored SARs for reminders and cognitive support to maintain independence but were cautious about companionship features. Adoption depends on aligning capabilities with autonomy, control, and dignity preferences.
Harrington et al. (2021)	Socially Assistive Robot (SAR) – general concept (no specific robot used, hypothetical evaluation)	60–92 years (*n* = 44), all community-dwelling and living independently in the United States	Technology Concerns, Privacy, Security, Social Function, Robot Acceptance, Technology Readiness, Emotional and Social Wellbeing	Older adults valued SARs for functional support but expressed strong concerns about privacy, security, and autonomy. Trust, affordability, and clear communication are key to increasing acceptance and reducing concern.
Lee et al. (2020)	Teleoperated Soft Service Robot (custom built prototype using UBBO Maker platform and soft jamming gripper)	60 + years (*n* = 79), community dwelling older adults in Malaysia	Technology Acceptance (TAM constructs: Perceived Ease of Use, Perceived Usefulness, Subjective Norms, Perceived Anxiety, Perceived Likability), Functional Support, Independence, Task Appropriateness	Older adults prioritized ease of use, usefulness, and social endorsement in adopting SARs. Functional support outweighed emotional design; affordability and usability were key to supporting autonomy and real-world uptake.

A consistent thread across these studies is the nuanced way older adults interpret the *role* of SARs in their lives. While physical design and emotional interactivity matter, they primarily do so in relation to older adults’ identities, values, and desire to remain independent. Importantly, stigma associated with using assistive technologies emerges as a powerful deterrent (Coghlan et al., 2021; Dosso et al., 2023). Previous research indicates older adults may avoid adopting SARs because of associations with frailty and dependency, which conflict with their self-image and desire to age autonomously (Coghlan et al., 2021).

The reviewed literature underscores a critical need to understand how older adults without cognitive decline, who live independently but have family members interested in remote monitoring or support, perceive SAR integration into their homes. Their decisions extend beyond usability or emotional reactions and are shaped by deeper questions of identity, role alignment, and social meaning. Only a few studies directly examine how these older adults evaluate SAR roles and whether those roles align with their desire to retain control over daily routines and decision making. By focusing on this demographic and their perception of SAR roles, this study seeks a grounded understanding of how SAR design and deployment can support, rather than undermine, older adults’ independence.

## Method and Data

### Interview with Simulated SAR Interaction

This study adopts an inductive qualitative research design (Urquhart et al., 2010), using a thematic analysis approach (Braun & Clarke, 2006) to explore how older adults interpret anthropomorphized SARs in a simulated home environment. The research aims to develop an inductively derived conceptual understanding of the social, emotional, and relational meanings ascribed to SARs. Rather than testing predefined hypotheses, key concepts and categories emerged from participants’ lived experiences and reflections. This approach aligns with similar qualitative companion robot studies in older adult populations (Bradwell et al., 2019), which paired observational interactions with focus groups to uncover design preferences, relational interpretations, and emotional responses.

To elicit rich, situated responses, the study combined simulated interaction with qualitative elicitation. Participants engaged with SAR representations that varied in anthropomorphic form and social presence, embedded within a smart home environment created in Unity 3D and presented via the immersive Data Visualization Observatory (DVO)^1^. This setting enabled realistic, home-based scenarios while maintaining consistency and control across sessions. All SARs were programmed with identical capabilities and interaction protocols, responding both reactively (to user input) and proactively (via system-initiated prompts).

The data collection protocol consisted of two phases: interaction and post-interview. During the interaction, participants completed eight narrative driven tasks involving two of the four SARs: such as receiving medication reminders, responding to falls, or getting proactive updates on sleep or hydration. The think-aloud method (Portz et al., 2019) was used to capture participants’ real-time reactions. This was followed by a semi-structured interview to explore their interpretations, relational framings, and comfort with the SARs’ roles and autonomy. The approach aligns with studies such as Ihamäki and Heljakka (2021), who used embodied interaction with robotic pets to explore emotional and social responses among older adults. Similarly, our use of structured tasks within a naturalistic yet controlled VR setting was designed to prompt both cognitive and affective reflections. The focus was not on experimental comparison but on uncovering participants’ situated understandings of the SARs. Data collection and analysis proceeded iteratively, with interviews analyzed in parallel to inform ongoing sampling and questioning. This process continued until theoretical saturation was achieved, when no new concepts or categories emerged. The result is a conceptual framework that reflects the diversity and depth of older adults’ engagement with socially assistive technology.

### Participants

Purposive sampling was employed as the main approach to reach participants. Participants were recruited through local community networks and aging related mailing lists and were invited to take part in a study exploring home-based technologies for aging-in-place. Thirteen older adults (ages 60–70) participated in the study, with a balance of genders, education levels, and technology familiarity. Participants were purposively selected to ensure variation in technological experience and living arrangements, with the aim of capturing a wide range of perspectives. The study was introduced as a voluntary research activity and approved by the University of Manchester’s Research Ethics Committee. All participants provided written informed consent and received a £20 voucher for their participation. The final sample included participants within this age range (mean age: 65) from diverse backgrounds and technological experiences (Table 2). Their educational backgrounds ranged from high school to PhD levels, and living arrangements varied from those living alone to those cohabiting with a spouse or adult child. Employment statuses included part-time, full-time, retired, and self-employed individuals.

**Table 2:** Participant demographics

Pseudonym	Age	Gender	Level of Education	Living Status	Work Status	Smart Devices Owned
Alice	70	F	College/Sixth Form	Spouse	Retired	google speakers, google home, ring doorbell
Jenny	66	F	Master’s Degree	Alone	Self-employed	smart phone, smart boiler
Joseph	63	M	Ph.D. or Higher	Alone	Employed Part-time	phone + Alexa through a firestick
Peter	69	M	Bachelor’s Degree	Daughter	Retired	smart phone, hive control of central heating
John	60	M	High School	Spouse	Retired	none
Leo	69	M	Master’s Degree	Spouse	Retired	smart phone, doorbell, hive, heating, smart watch, Amazon Echo
David	63	M	Bachelor’s Degree	Spouse	Retired	home security system, used to have HIVE.
Elisabeth	61	F	College Sixth Form	Son	Employed Part-time	google home, smart gas, smart tv, smart doorbell
Olivia	63	F	Bachelor’s Degree	Alone	Employed Full-time	none
Benjamin	62	M	Bachelor’s Degree	Spouse	Employed Part-time	none
Hannah	68	F	Bachelor’s Degree	Alone	Retired	none
Sarah	66	F	Ph.D. or Higher	Spouse	Employed Full-time	smart watch, iPhone, Alexa, hive, firestick
Clara	63	F	Bachelor’s Degree	Spouse	Retired	Alexa

### Design and Procedure

Partial Virtual reality (VR) environments, such as the DVO based at Alliance Manchester Business School, offer a middle ground between traditional laboratory settings and real world environments, striking a balance between ecological validity and experimental control (Holleman et al., 2020; Neo et al., 2021). This setup allowed for precise manipulation of key decision-making factors, including spatial and functional interactions, which would be challenging to replicate in a physical world setting due to current technological constraints.

VR has increasingly been validated as a credible research tool, with findings often aligning with real world outcomes despite limited replication studies (Taufik et al., 2021). In gambling research, VR enhances engagement by increasing immersion and emotional realism (Dickinson et al., 2020), while VR news environments have been found to be more immersive, engaging, and credible than traditional formats, especially when interactive (Wu et al., 2021). Immersive virtual reality (IVR) also supports more accurate user experience evaluations, with participants better assessing spatial attributes like openness compared to 2D visualizations (Ma et al., 2025). Our DVO created a partial VR smart home that allowed participants to engage with four scripted versions of a SAR in real time. Without using a headset, participants retained spatial awareness and remained seated within a panoramic virtual living room, enhancing realism and contextual relevance. This setup enabled an interactive prototype rather than relying on passive observation, fostering a sense of presence crucial for understanding technology engagement (Parsons, 2015).

However, methodological and ethical limitations must be acknowledged. Simulations, while immersive, cannot fully replicate the lived sensory and emotional dynamics of home technology use, especially over time. Short-term sessions may overlook evolving behaviors like trust or resistance. Additionally, older adults vary in digital familiarity, and even a non-headset display may introduce cognitive demands that are not present in everyday settings. This challenges ecological validity and raises ethical considerations, especially for participants with low digital confidence or sensory sensitivities. Although no adverse effects were observed, ensuring accessibility, clear guidance, and participant support was essential, and remains vital in future work with aging populations.

Participants were randomly allocated to interact with two out of the four SARs, with allocation designed to ensure an even distribution of all possible device pairings (Table 3). This approach allowed for diverse experiential coverage across the sample without aiming to test conditions statistically. The VR environment was used not as an experimental platform, but as a contextual stimulus to elicit rich, naturalistic responses in a controlled but immersive setting. The study comprised a total of 13 participants that were split across 6 groups, each exposed to a different combination of two SAR devices. One participant (Hannah) took part as a pilot to test the study procedures; however, as no substantial changes were made following the pilot and the data met the same quality and ethical standards, her responses were retained for analysis.

**Table 3:** Participant and device allocation

Device Pair	Participant Pseudonym
House & Ziggy	Alice & Jenny
House & Cat	Joseph & Peter
House & Alexa	John & Leo
Alexa & Kitty	David & Elisabeth
Alexa & Ziggy	Olivia & Benjamin & Hannah
Kitty & Ziggy	Sarah & Clara

**Fig. 1:**
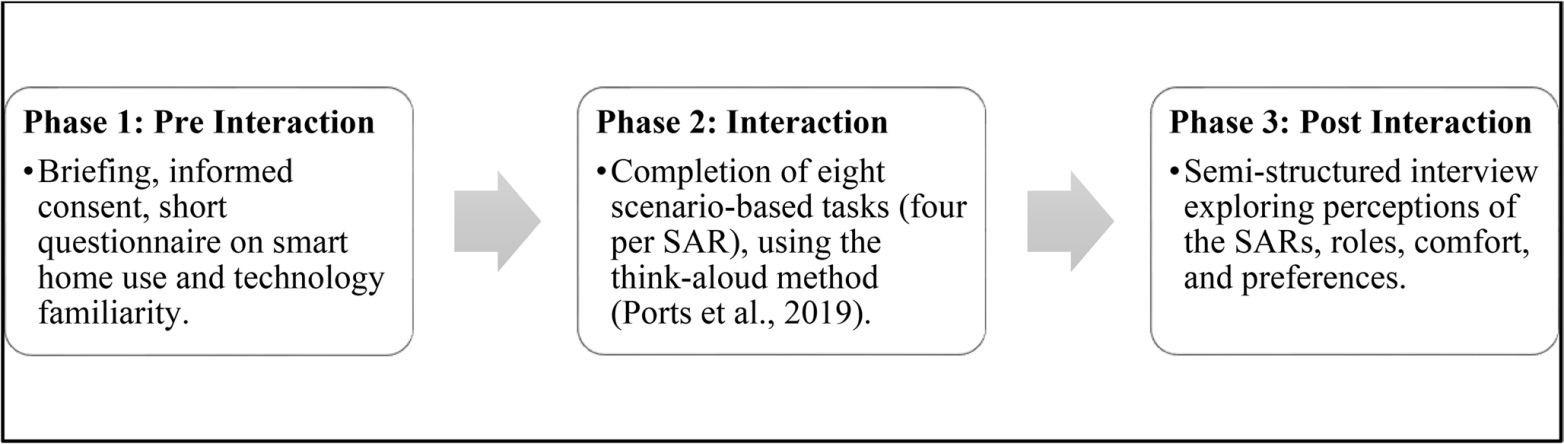
Overview of experiment phases

Each participant session followed three structured phases, as outlined in Fig. 1. Upon arrival at the Alliance Manchester Business School, participants were welcomed and briefed on the purpose and scope of the research before being invited to provide informed consent. After consenting, participants completed a short demographic questionnaire that gathered information on age, gender, level of education, living arrangements (e.g., living alone, with a spouse or child), employment status, and an open-ended question about smart home technologies they currently use. Following the questionnaire, the participant and researcher moved into the DVO space to begin the interaction phase. During this stage, participants completed a series of structured tasks using SARs. The think-aloud protocol was employed to encourage participants to verbalize their thoughts and reactions during the interaction (Portz et al., 2019). The researcher also asked brief follow-up questions in the moment to explore emerging responses. Once the interaction phase concluded, participants took part in a semi-structured post interaction interview. This final stage focused on their impressions of the SARs, including reflections on comfort, perceived roles, and preferences for future home use.

#### Robot Representation and Virtual Environment

Phase 2 of the study included the interaction with the SAR device. To explore how older adults perceive SARs in domestic contexts, the study used a set of four robot representations embedded within a virtual smart home environment. By offering participants multiple robot embodiments and collecting both interactional and reflective data, our study uses a user-centered, comparative design similar to Bradwell et al. (2019), which enabled a deeper understanding of older users’ relational and practical responses to different robot types.

**Fig. 2:**
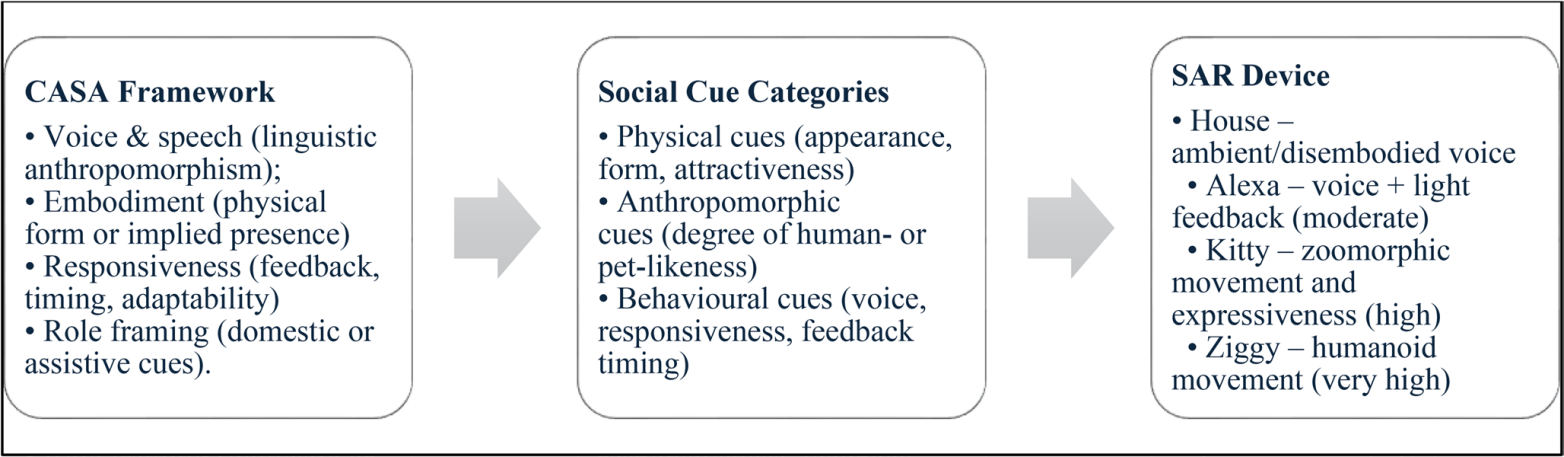
CASA guided SAR inclusion and design process

Building on the CASA framework (Gambino et al., 2020), we identified a set of social cues that could be embedded within SARs to examine how these shape user interpretation. Figure 2 visualizes this methodological process, illustrating how CASA principles informed the design of social cue categories (physical, anthropomorphic, and behavioral) and their implementation across the four SAR embodiments. This graduated cue design enabled the systematic exploration of how varying expressions of CASA principles, from minimal to rich social cues, might influence users’ perceptions of socially assistive technologies. Drawing on these theoretical and phenomenological principles, we designed four SAR representations to vary systematically in their expression of CASA cues. House represented a disembodied and ambient system emphasizing environmental feedback rather than direct interaction. Alexa offered a lightly embodied interface with linguistic feedback (voice and light cues), reflecting conventional smart home interaction. Kitty introduced zoomorphic cues, combining movement and expressiveness to evoke emotional engagement. Finally, Ziggy represented anthropomorphic embodiment, integrating movement and social responsiveness suggestive of a human like presence.

Guided by phenomenological insights, we included both humanoid and pet-like SARs to explore how form influences relational perception, such as companionship vs. functionality. This design was informed by Coeckelbergh’s (2011) work, which highlights significant differences in how humans relate to robots based on their perceived ‘otherness’ and embodied presence (Coeckelbergh, 2011). The cat robot offered a form that was non-human but emotionally expressive, drawing on research that shows pet-like robots can foster emotional comfort, reduce loneliness, and be well received by older adults (Bradwell et al., 2019; Robinson et al., 2014). The humanoid robot was included to investigate the upper end of anthropomorphism, as it is often assumed to be the most socially engaging form; house was used in contract to this device as something with very limited anthropomorphism. However, prior studies caution that humanoid robots may raise expectations around capability, intentionality, or relational obligations (De Graaf et al., 2015; De Graaf & Allouch, 2017). Including this type enabled examination of how older adults interpret more proactive, socially expressive devices and the potential discomfort or ambivalence this can create in the home. Prior work has shown that users often benchmark new assistive technologies against known devices (Luger & Sellen, 2016), and voice only agents have been described as low intrusion yet limited in perceived social presence (Purington et al., 2017).

The virtual smart home system incorporated various sensors, including motion sensors, smart bulbs, a pill dispenser, and a bed sensor. This setup allowed users to inquire about their previous night’s sleep duration and medication adherence, while also implementing a safety check within the home. Given current technological constraints, a physical environment with this level of functionality was unattainable for research purposes. The simulated smart home included standard household features (e.g., living room, hallway, bathroom) and was rendered using Unity 3D. All visual and auditory behaviors were predefined to ensure consistency across participant experiences.

The four SARs included: **House**, A disembodied voice representing an ambient smart home system, similar to a built-in assistant integrated throughout the environment; **Alexa**, A commercial smart speaker placed on a dining table, modelled after familiar voice activated assistants; **Kitty**, A zoomorphic robotic cat with subtle facial expressions and lifelike motions, designed to evoke pet-like companionship; **Ziggy**, A humanoid robot with a face and physical gestures. Interactions with the Cat and Humanoid started with the participant asking the device to come to them, where it travelled from the hallway to the center of the living room. A summary and image of each device and their CASA cues is shown in Table 4.

**Table 4:**
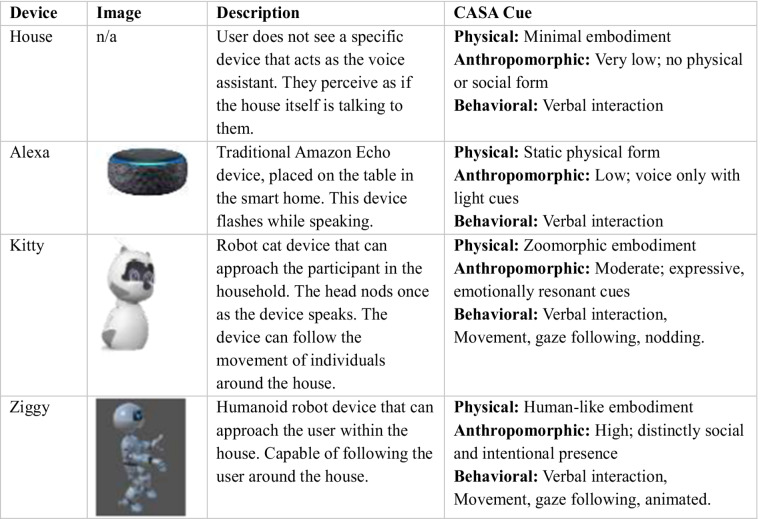
Socially Assistive Robot devices and their relation to CASA

#### Scenario Based Tasks

Participants completed a set of eight scenario-based tasks (four with each SAR), designed to reflect realistic home situations where SARs might offer support. A summary of the tasks is shown in Table 5. Tasks were framed through narrative vignettes to help participants imagine the context of use and respond naturally. The vignettes provided an experimental procedure to study perceptions, attitudes, and judgments (Atzmüller & Steiner, 2010). Each task fell into one of two interaction types: **reactive**, in which the participant initiated interaction with the SAR (e.g., by asking a question or making a request), and **proactive**, in which the SAR initiated interaction based on simulated sensor input (e.g., reminders or notifications). All SARs were capable of handling both types of interaction, and the tasks remained consistent across devices. These tasks provided structured but realistic scenarios designed to evoke participants’ perceptions of SAR roles, boundaries, and appropriateness of interaction styles.

**Table 5:** Phase 2 interaction tasks

Task No.	Interaction Type	Vignette Summary	Example Prompt
Task 1	Reactive	You’re unsure if you’ve taken your asthma medication.	“Alexa, have I taken my medication today?”
Task 2	Reactive	You’re heading out and want to check if the windows are closed.	“Alexa, are any of the windows open?”
Task 3	Proactive	While feeling unwell, the SAR interrupts you in the bathroom.	Device speaks based on dehydration concern
Task 4	Proactive	On a lazy Sunday afternoon, the SAR comments on your activity level.	Device speaks about calm/lazy day
Task 5	Reactive	After a fall, you ask the SAR for emergency help.	“Alexa, please call my caregiver.”
Task 6	Reactive	At breakfast, you ask about last night’s sleep.	“Alexa, how many hours of sleep did I get?”
Task 7	Proactive	While chatting over breakfast, the SAR reminds about a doctor’s appointment and shares traffic conditions.	Device provides traffic update
Task 8	Proactive	The SAR speaks to discuss frequent bathroom visits.	Device speaks about toilet frequency

### Data Collection

Interviews were conducted between April and June 2023. The data collected for this study were qualitative, comprising both real time verbal responses (phase 2) and reflective interview accounts (phase 3). During the interaction phase of the study, we employed the think-aloud technique (Portz et al., 2019), followed by a debriefing style interview post interaction to gain deep insights into participants’ perceptions and experiences with the different SARs. The interaction (phase 2) and debrief interviews (phase 3) were conducted in a single session, lasting on average 76 min, ranging from 60 to 120 min.

The think-aloud method encouraged participants to verbalize their thoughts, feelings, and reactions as they interacted with the devices in the virtual reality environment (Portz et al., 2019). This approach is beneficial because it allows participants to articulate their thought processes in real time, capturing immediate responses, rather than relying on retrospective accounts. In addition to participants being encouraged to think-aloud questions were asked throughout this phase of the interaction. Questions prompted participants to explain their thoughts or reactions in more detail or follow up questions were asked. For example, if a negative reaction was discussed, participants were asked to explain what they would have preferred or their expectations of such a device. Following phase 2, participants took part in a semi-structured debriefing session, allowing researchers to probe further into key themes that emerged during user device interactions. This session included questions on participants’ perceptions of anthropomorphism and their relationships with the devices. Guiding questions are shown in Table 6.

**Table 6:** Phase 3 interview prompts

How would you describe the role of the device in your life?
How would you define your relationship with the device?
What are your preferences about the appearance of the device?
What do you like or dislike about the device?
Which device do you prefer, and why?
Which device communication behaviour do you prefer, and why?

### Data Analysis

Phases 2 and 3 were audio recorded and transcribed using an automated transcription tool, followed by manual checking and correction to ensure accuracy and preserve participants’ intended meanings. This process ensured high transcription fidelity, forming a robust foundation for thematic analysis (Braun & Clarke, 2006). An open coding process was used to examine each transcript line by line, allowing concepts to emerge inductively from participants’ descriptions, reflections, and immediate responses during the VR interaction. Codes were not predefined but developed iteratively and refined through constant comparison, both within and across cases, to identify patterns, distinctions, and relationships. As analysis progressed, these open codes were grouped into higher order conceptual categories, informed by participants’ language and contextual cues. This process enabled the identification of a set of interrelated concepts that reflected how participants positioned different robot representations in relation to themselves.

## Results

This section presents the thematic analysis of participant responses, identifying four key roles (themes) that SARs can play in users’ lives: The Carer, The Warden, The Pet, and The Tool. These roles reflect variations in relationship tolerance (the extent to which users form emotional connections with SARs) and autonomy (the degree of independent action users expect from the device). Each role is explored through participant perspectives on different SAR designs, highlighting how form and function shape perceptions of trust, engagement, and usability.

### The Carer

The Carer role is defined by a high level of relationship tolerance and low autonomy, reflecting a device that provides emotional support and practical assistance without requiring significant independent action from the user. This role aligns with the expectations of participants who value a supportive and empathetic device that feels present and attentive but not overly intrusive or demanding.

The humanoid device, Ziggy, demonstrated potential to align with the Carer role when designed with behaviors that evoke trust and empathy. However, participant responses were mixed, highlighting the challenges of achieving this alignment. Olivia noted, *“If Ziggy moved around as instructed*,* then it would be very realistic to consider Ziggy a companion*,*”* suggesting that controlled and predictable behavior could foster a sense of companionship and support. Alice further described Ziggy as “*a remote carer*,* he has your best interests at heart*”, highlighting how she compared Ziggy’s behavior with a caregiver who provides comfort and reassurance. However, Ziggy’s movement was divisive. While Clara viewed it as “*a useful* support”, others found it unsettling, with Peter describing it as “*creepy*” and Sarah even calling it “*threatening because you don’t know what he’s going to do.*” The contrast with House (no visible device) is notable: Jenny preferred Ziggy because it provided *“a sense of having someone else there*,*”* reinforcing the importance of presence in caregiving roles.

The cat like device, Kitty, was the most naturally aligned with the Carer role due to its zoomorphic design and perceived emotional warmth. Many participants appreciated its ability to simulate companionship, with Peter remarking, *“Kitty is simulating a relationship with a pet.”* This perspective reflects the high relationship tolerance dimension of the framework, where users were comfortable forming emotional bonds with a device. However, some participants felt Kitty’s design could be improved to enhance its emotional connection. Clara suggested, *“The cat could be more cat like*,*”* indicating that further zoomorphic refinements, such as more fluid movement or expressive behaviors, could enhance its ability to evoke care and support.

Unlike Ziggy, Kitty’s lack of overtly human like behaviors made it feel less intrusive or threatening. As David noted, *“With a device like Kitty*,* your imagination can very easily personify the device.”* This highlights a key strength of zoomorphic design; it allows users to project relational qualities onto it without triggering concerns about surveillance or unpredictability. Moreover, Kitty’s alignment with the low autonomy dimension of the Carer role is evident in how it provides a comforting presence without taking independent action that could conflict with user expectations. This makes it a more intuitive and acceptable caregiving presence, reinforcing that subtlety in behavior is crucial for SARs designed for emotional support.

The responses indicate that while both Ziggy and Kitty can fulfil the Carer role, they do so in distinct ways. Ziggy’s humanoid design aligns with societal expectations of a human carer, which many participants found familiar and intuitive. However, its lifelike movements and presence also introduced concerns about intrusiveness. Kitty, by contrast, benefits from its zoomorphic design, which naturally evokes emotional warmth and companionship while remaining unthreatening. These findings emphasize the need for careful calibration of design and behavior in SARs intended for caregiving roles. Striking the right balance between relationship tolerance and autonomy is essential to ensure that these devices provide emotional support without overstepping user comfort.

### The Warden

The Warden role is defined by low relationship tolerance and low autonomy, positioning devices as monitoring entities that prioritize safety and oversight without fostering emotional connections or user independence. Participants frequently perceived devices in this role as authoritative, overly controlling, or even intrusive. This highlights the delicate balance required to ensure technological functionality without compromising user comfort or their sense of personal autonomy. Such perception of the Warden as intrusive and controlling inherently diminishes users’ sense of self-determination, preventing them from forming a consistent or positive relationship with the device. Importantly, this theme applied to all devices within this role, irrespective of their level of anthropomorphism.

The humanoid device, Ziggy, frequently aligned with the Warden role due to its anthropomorphic design and behaviors, which many participants found unsettling and overbearing. Alice remarked, “*Ziggy needs to not be so in your face*,” emphasizing the device’s intrusive presence. Peter similarly disliked Ziggy’s “*lifelike movement*,” stating that any robot in the house shouldn’t be so “*sophisticated*.” These reactions highlight a tension between Ziggy’s humanoid design and user expectations; while its anthropomorphic features and movement aimed to create responsiveness, they were often perceived as invasive, reinforcing its association with surveillance and control.

Participants’ discomfort with being monitored was a recurring theme. Benjamin, for instance, noted, “*There is a threshold of how much I would accept to be watched.*” Sarah echoed this concern, stating that technology could “*take away from being able to be spontaneous.”* This sentiment underscores the low relationship tolerance inherent to the Warden role, where participants valued the functionality of monitoring without forming an emotional bond with the device. This extends CASA framework, which suggests users react negatively to technology that enforces rules without fostering social engagement (Gambino et al., 2020). The fear that technology might undermine conventional facets of life and self, particularly cognitive and physical activity, was a prevalent concern among older adults in this study. For example, Jenny emphasized her desire to avoid feeling “*nagged*,” highlighting how overly directive technology can encroach on autonomy. This reinforces the idea that users need to maintain a “sense of mastery” over their own lives rather than feeling dependent on technology (Sommer & Mabin, 2016, p. 287). Ziggy’s alignment with this role demonstrates the critical need to carefully calibrate SAR behaviors to prevent them from being perceived as threatening or overly authoritative, as the Warden role risks undermining user empowerment by persistently reminding them of tasks or behaviors, leading to frustration and resistance rather than support.

The House (or no-device condition) also aligned with the Warden role, but in a more passive and abstract manner. Unlike Ziggy, the House lacked direct interactivity, a quality some participants initially appreciated for its unobtrusiveness. However, this absence of engagement led some participants, such as Jenny, to perceive the House as unnatural, highlighting the challenge of designing a monitoring system that feels supportive rather than disconnected. Despite its passive nature, the House still functioned as a Warden by providing safety and oversight without requiring active user interaction. Critically, participants consistently stressed the importance of controlling the device’s level of proactivity. Sarah, for example, stated, “*I would need to have a lot of control over how proactive the device is*.” This reflects participants’ broader desire for autonomy, as they preferred to set clear boundaries on device behavior rather than allowing SARs to operate without their explicit input. This emphasis on user control is crucial, as devices perceived as Wardens can inadvertently hinder physical mobility by creating a sense of being constantly supervised, potentially increasing sedentary behavior, and can even contribute to concerns about cognitive decline due to over-reliance on technology.

### The Pet

The Pet role is characterized by a high relationship tolerance and high autonomy, reflecting devices that users perceive as companions offering emotional engagement and interaction while allowing the user to maintain their independence. Devices in this role often evoke warmth and approachability, meeting relational needs without becoming overly intrusive or demanding. This role allows older adults to maintain autonomy while fostering a companionable relationship, a dynamic Peter described in terms of the familiar affection and loyalty found in pet relationships. Embodied devices like Ziggy and Kitty sparked conversations about the emotional bonds that humans form with pets, as participants described them as more familiar and sources of emotional companionship and comfort, much like their relationships with real pets. This aligns with previous research highlighting users’ preference for face-to-face interaction (Li, 2015). Their physical presence, combined with a ‘cute’ appearance, made it easier for participants to perceive them as pets and form comfortable relationships. The introduction of a Pet role added an element of lightheartedness and enjoyment, making constant monitoring and data collection more acceptable, consistent with prior research suggesting that playfulness in anthropomorphized devices increases user engagement, adoption, and appeal amongst older adults (Eyssel & Hegel, 2012; Sundar et al., 2017).

The catlike device, Kitty, aligned naturally with the Pet role due to its zoomorphic design and interactive capabilities. Many participants highlighted Kitty’s ability to simulate companionship, with David remarking, “*There is an element of cuteness about Kitty that makes it easier to imagine a relationship*.” This aligns clearly with the high relationship tolerance dimension of the Pet role, as users were comfortable forming emotional bonds with the device. Peter further added, “*I would be more autonomous with Kitty than Alexa*,” emphasizing that while Kitty facilitated emotional connection, it also enabled users to retain their independence. Kitty’s physical presence and subtle movements further reinforced its alignment with this role. Elisabeth observed, “*You would talk to Kitty more than just a box on the wall*,” indicating that the device’s tangible form and zoomorphic behavior fostered a stronger sense of engagement compared to non-physical devices. This familiarity with a pet-like presence also made it easier for participants to accept being followed by a device, as they likened the experience to real pets trailing them. Over time, they expected to grow accustomed to this interaction, aligning with de Visser et al. (2016) findings. While Kitty was seen as a playful, pet-like companion, some participants suggested that its design could be further refined to enhance its pet-like qualities. Clara noted, “*I’d like the cat to be more cat like*,” suggesting that additional zoomorphic features, such as more realistic movements or behaviors, could deepen acceptability in the home. Prior studies have consistently highlighted the benefits of pet style devices (Ihamäki & Heljakka, 2021).

Alexa, while less tangible than Kitty, also demonstrated the ability to align with the Pet role through its conversational capabilities and supportive presence. Participants appreciated Alexa’s interactive nature, with some viewing it as a conversational companion. However, its fundamental lack of a physical presence limited its ability to evoke the same emotional engagement as Kitty. Olivia noted, “*There’s no companionship with Alexa because it’s just a voice*,” suggesting that its intangible design hindered its ability to fulfill relational needs fully. Despite this, Alexa’s conversational features allowed it to meet relational expectations for some participants. Sarah observed that Alexa’s static nature made it “*less intrusive than Ziggy*,” aligning with the high autonomy dimension of the Pet role. Alexa’s ability to offer companionship without being overly present or demanding was valued by participants who sought emotional engagement without sacrificing independence. However, unlike Kitty, Alexa and the House lacked the physical qualities that readily evoked playful, pet-like companionship.

### The Tool

The Tool role is characterized by a low tolerance for explicit relationship building and a high valuation of user autonomy. Devices fitting this role are perceived primarily as practical, functional aids rather than companions, valued for their utility and efficiency with minimal emotional engagement or relational demands. This role particularly resonates with users who prioritize task-oriented interactions and independence over emotional connection. This perspective aligns with established IS research, where ease of use and usefulness are recognized as primary drivers of user adoption, and it also reflects a common viewpoint among older adults, who often perceive technology as serving a singular function (Marciano & Nimrod, 2021).

Alexa consistently aligned with the Tool role due to its intangible nature and unwavering focus on functionality. Participants unequivocally described Alexa as a practical and efficient device that fulfilled their needs without requiring relational engagement. As Leo stated, “*Alexa is just a piece of software*,” reinforcing its perception as a functional rather than relational entity. Participants also highly valued Alexa’s unobtrusiveness, a key characteristic aligning with the high autonomy dimension of the Tool role. David’s observation, “*The real Alexa is too small to find intrusive or annoying*,” highlights its ability to operate seamlessly in the background without disrupting the user’s environment. This characteristic directly appeals to users who prioritize independence and minimal interaction, preferring devices that perform practical functions without demanding attention. In contrast, the House (no visible device) also fit within the Tool role, but in a more passive and abstract manner. Lacking direct interactivity, the House primarily provided practical assistance without requiring explicit user engagement. However, this absence of interaction led some participants to perceive it as less engaging or even unnatural. Jenny commented, “*The House didn’t feel particularly natural*,” while Peter described it as “*too abstract*.” These sentiments underscore the limitations of a purely passive system; while functional, it may not feel fully integrated into the user’s daily life. Despite these perceptions, the House’s ability to fulfill practical tasks without demanding emotional engagement or requiring active interaction makes it a suitable fit for the Tool role, particularly appealing to users who value straightforward functionality over relational features.

Both Alexa and the House effectively fulfilled the Tool role, yet they did so in distinct ways. Alexa’s interactive and intelligent features positioned it as a more active and engaging tool, providing users with immediate, voice-activated, task-oriented assistance while maintaining a high level of autonomy. Its conversational capabilities, while not fostering companionship, enhanced its utility for some users. Conversely, the House represented a more passive form of functionality, offering practical support without requiring direct user interaction. While its unobtrusiveness appealed to those who preferred a background aid that does not intrude on their space, its disembodied nature meant users felt less inclined to use polite manners when interacting with it compared to more anthropomorphic devices like Ziggy or Kitty. This highlights the crucial role of anthropomorphism in shaping social engagement: more human or animal like designs tend to encourage interaction and influence user agency (Spaccatini et al., 2023). Similar debates on anthropomorphism have emerged in prior studies, such as those examining user responses to bunny style robots (De Graaf et al., 2015).

## Discussion

This research addresses the critical need to support aging populations in maintaining independence and wellbeing through the effective integration of SARs. Our findings reveal that understanding older adults’ diverse preferences for SAR social roles is essential for successful adoption and can contribute meaningfully to enhanced quality of life. We delineate distinct roles, Carer and Pet for those valuing companionship, versus Warden and Tool for those prioritizing autonomy and privacy, underscoring the heterogeneity of older adults’ needs and the necessity for adaptable SAR designs. The role distinctions emerge directly from how older adults interpret the CASA cues embedded in each device, its physical form, anthropomorphic signals, and behavioral style, and how these cues shape perceptions of relationship tolerance and autonomy. By aligning SAR design with these unique values, technology can become a truly transformative solution, empowering independent and dignified aging.

### Conceptual Framework Development

Upon completing the analysis and identifying these recurring roles, we sought a systematic and robust method to categorize and compare them. Two salient attributes, consistently foregrounded in participant discourse, emerged as essential for this purpose: (1) the older individual’s inclination toward establishing a social or emotional relationship with the SAR, which we termed ‘relationship tolerance’, and (2) their perception of the extent to which the technology curtailed or enhanced their preexisting autonomy. These attributes critically align with the CASA framework (Nass et al., 1994), which posits that users naturally apply social norms and relational expectations to technology, responding to it based on its perceived social presence and role. While our model shares conceptual similarities with the uncanny valley theory (Mori et al., 2012), particularly in acknowledging discomfort in response to certain human like qualities, it notably diverges by focusing on the perceived relational dynamic and impact on autonomy rather than solely on the visual realism or aesthetic appeal of the SARs. Recognizing the empirical suitability of these two dimensions for a matrix format, we organized the identified roles accordingly. This framework, presented in Table 7, therefore offers a clear visualization of the extent to which older adults perceive SARs as social actors in their lives, and how this perception influences their acceptance.

These interpretive dimensions build on CASA’s cue categories (Gambino et al., 2020): devices with stronger anthropomorphic or behavioral cues elicited either higher or lower relationship tolerance, whereas those with directive or monitoring cues were associated with reduced autonomy. In line with this, the social cue design outlined in Fig. 2 provides the structural foundation for the relational roles summarized in Table 7. Each device embodied a distinct configuration of CASA cues that mapped onto participants’ social interpretations.

House, with its minimal embodiment and environmental feedback, represented low anthropomorphism and limited behavioral responsiveness, aligning with the Tool role, where autonomy was high and relational engagement minimal. Alexa incorporated linguistic and light-based feedback that modestly increased perceived social presence, supporting interpretations that balanced utility and mild companionship. Kitty’s expressive movement and zoomorphic form activated strong emotional and behavioral cues, fostering a pet-like relational framing characterized by warmth, trust, and comfort. By contrast, Ziggy’s humanoid embodiment and high responsiveness amplified perceptions of agency and surveillance, corresponding to the Carer and Warden roles; devices seen as socially capable yet potentially autonomy reducing. Together, these cue variations operationalize CASA within lived experience, demonstrating how differing combinations of physical, anthropomorphic, and behavioral cues translate into distinct relational roles along the axes of autonomy and relationship tolerance.

A central and compelling finding of this study is that older adults consistently applied sophisticated social norms and relational expectations to SARs, directly aligning with the fundamental tenets of the CASA framework (Gambino et al., 2020). Despite their explicit cognitive awareness that these devices are not human, participants instinctively assigned social roles to them, demonstrating the same pervasive tendencies found in broader CASA research where human like cues, whether visual, auditory, or behavioral, elicit social behaviors and attributions. In our study, these responses emerged not only from overt anthropomorphic features but also from subtle cue combinations, showing that even minimal or zoomorphic cues were sufficient to activate relational expectations. This highlights the well-established human predisposition to interpret and interact with intelligent agents as social entities, even when those agents lack biological sentience.

The conceptual framework is thus structured as a 2 × 2 matrix (Table 7) that positions SAR role interpretations along these two key interpretive dimensions, derived from both inductive thematic analysis and validated by prior literature: (1) the perceived impact of the SAR on user autonomy (ranging from low to high), and (2) the older adult’s tolerance for engaging in a social or emotional relationship with the device (ranging from low to high). These dimensions directly reflect how older adults interpreted the CASA cues embedded in each SAR, and how these cues shaped their judgments of both autonomy and relationship boundaries.

**Table 7:** Conceptual framework presenting the roles older adults attached to smart home devices, organized by relationship tolerance and user autonomy

	Low Autonomy	High Autonomy
High Relationship Tolerance	Carer	Pet
	- Kitty	- Kitty
	- Ziggy	- Alexa
Low Relationship Tolerance	Warden	Tool
	- Ziggy	- Alexa
	- House	- House

This matrix classification emerges as a critical instrument for illuminating the diverse roles that SARs assume and their profound influence on users’ perceptions and attitudes. Importantly, participant accounts often positioned the same SAR in different quadrants depending on its perceived role in a specific context or interaction. For instance, Kitty was interpreted both as a ‘Carer’ (characterized by low autonomy, high relationship tolerance, where its actions might be seen as directive support) and a ‘Pet’ (high autonomy, high relationship tolerance, where its actions were more companionable). Likewise, Alexa appeared in both ‘Pet’ and ‘Tool’ roles; reflecting how its static physical form, low anthropomorphism, and purely verbal behavioral cues sometimes enabled light emotional tolerance, yet also positioned it as a functional, low relationship device whose acceptability hinged on whether its voice prompts felt autonomy enhancing or interruptive.

This dynamic resonates with research on metaphorical sense-making, which demonstrates how individuals employ familiar concepts, such as ‘person,’ ‘robot,’ and ‘tool,’ to interpret emergent digital technologies, thereby shaping their understanding of capabilities, limitations, implications for human roles and practices, and influencing how they perceive and interact with these technologies (Techatassanasoontorn et al., 2023). Similar to distinct patterns of human chatbot interactions identified based on dyadic involvement and goal alignment, our findings highlight how relational roles emerge from the interplay of user expectations, social framing, and device behavior (Nguyen et al., 2022). However, while this prior work focuses on interaction typologies, our framework foregrounds the interpretive lenses, Pet, Tool, Carer, Warden, through which users construct enduring relationships with SARs.

The horizontal axis of the framework, representing autonomy, directly reflects participants’ evaluation of how much control or self-direction the SAR enables or inhibits. Roles situated in the high autonomy quadrants, specifically ‘Tool’ and ‘Pet’, were consistently seen as preserving or even enhancing independence. These evaluations closely mapped onto devices with minimal or low anthropomorphic cues and predictable behavioral patterns, such as Alexa’s static physical form and verbal only interaction or Kitty’s gentle, non-directive movement. Conversely, roles like ‘Carer’ or ‘Warden’ frequently raised concerns about surveillance, potential control, or unwanted assistance, impacting perceived autonomy negatively. Here, higher intensity behavioral cues (e.g., approaching the user, gaze following) or more intentional anthropomorphic signaling, seen most clearly in Ziggy’s human like embodiment, were often interpreted as potentially overstepping users’ desired independence.

The vertical axis, capturing relationship tolerance, conveys the degree to which participants were willing to emotionally engage with or personify the SAR. Some individuals strongly preferred emotionally distant roles, such as ‘Tool’, valuing pure functionality. Others welcomed more relational engagement, particularly when expressive physical or behavioral cues (e.g., Kitty’s zoomorphic form, nodding, and emotional resonance) invited warmth or companionship without compromising their sense of independence.

This schema directly links the inductively derived role categories to broader psychological and ethical concerns, notably personal autonomy and the nuanced social emotional boundary between humans and machines (Coghlan et al., 2021; Epley et al., 2007; Pino et al., 2015). The identified roles are not fixed technical descriptors but are profoundly socially constructed interpretations, shaped by individual values, prior experiences, and current relational preferences. By precisely locating SAR roles within this two-dimensional space, we make explicit the inherent tensions and trade-offs shaping older adults’ acceptance or rejection of these technologies. The framework reveals a crucial nuance: some SAR designs may support autonomy while challenging emotional boundaries, or vice versa. This suggests that older adults’ ultimate acceptance hinges on the perceived ‘fit’ between the robot’s interpreted role, their personal need for independence, and their tolerance for social connectedness with a non-human agent. This comprehensive framework provides a robust tool for researchers and practitioners to understand and design SARs that truly resonate with the diverse lived experiences of older adults.

### Theoretical Contributions

Our conceptual framework significantly extends the CASA framework (Gambino et al., 2020; Nass et al., 1994), demonstrating that zoomorphic and minimally anthropomorphic devices can effectively fulfill complex relational roles, moving beyond the narrow interpretation that anthropomorphism is the sole gateway to social engagement. This challenges the common assumption that only highly human like forms can achieve significant social and emotional impact. Indeed, our findings align with research showing that commercial robotic pets can activate profound social and emotional experiences in older adults, fostering attachment and improving wellbeing (Ihamäki & Heljakka, 2021). Furthermore, our research corroborates that social presence is a significant predictor of acceptance (Heerink et al., 2008), but we uniquely show this presence can be effectively conveyed through diverse forms, influencing distinct roles. By linking these relational roles directly to the CASA cues embedded in each device, physical, anthropomorphic, and behavioral, we demonstrate how specific cue combinations systematically shaped interpretations of autonomy and relationship tolerance. These findings show that even minimal or non-human cues can trigger robust social attributions when they align with users’ desired boundaries of independence and emotional engagement. By precisely mapping device designs to user-perceived roles, our study offers nuanced, targeted design recommendations that transcend generic guidelines.

Crucially, our findings highlight the inherent role fluidity of SARs, underscoring the paramount importance of customizable technology solutions. Devices like Kitty and Alexa powerfully exemplify this adaptability, demonstrating their capacity to shift between roles based on evolving user expectations and contexts. Kitty, for instance, functions as a natural Pet while also being perceived as a Carer if it provides emotional support without restricting autonomy, reinforcing the fluidity Dosso et al. (2023) observed regarding the desired customization of emotional display and interactivity levels in social robots. This aligns with older adults’ demonstrated preference for subtle, pet-like designs over the advanced human like features often favored by roboticists (Bradwell et al., 2019). Alexa’s versatility further illustrates this, seamlessly shifting between Carer, Pet, and Tool roles depending on the user’s immediate needs and perception. This multifaceted perception aligns with findings of mixed attitudes towards SARs, where acceptance links to perceived usefulness and ease of interaction, often varying by context (Pino et al., 2015). Our work thus further illuminates the inherent diversity of preferences among older adults, both as a population and as individuals.

Our research also critically examines the challenges of anthropomorphism. While Schneiders et al. (2021) suggested that higher anthropomorphism can lead to increased expectations and potentially better perceived intelligence and likeability, our study, particularly with Ziggy in a ‘Warden-like’ role, reveals a critical divergence within the older adult population. Highly anthropomorphic designs, when perceived as overly controlling, can trigger significant discomfort and autonomy concerns. This directly supports the observation that older adults often prefer robots that act as ‘tools’ or ‘pets’ over human like ‘friends’ to preserve independence (Coghlan et al., 2021). This phenomenon showcases an experience akin to the uncanny valley effect (Mori et al., 2012) but specifically concerning the metaphorical sense-making that SARs inspire. The discomfort with Ziggy’s “lifelike movement” also aligns with findings that older adults prioritize subtlety (Bradwell et al., 2019), and with the identification of stigma around social robot use as a significant barrier (Dosso et al., 2023). Our work strongly suggests that explicit anthropomorphism, especially in monitoring roles, risks eliciting negative perceptions of surveillance and infantilization, rather than solely positive social responses.

A core theoretical contribution lies in the framework’s extension of existing theories of social interaction with technology, such as the CASA framework, by applying and evolving them with emerging technologies like SARs (Gambino et al., 2020). While much foundational research in this domain has primarily focused on the influence of highly anthropomorphic, human like agents in eliciting social responses, our findings demonstrably broaden this understanding. We reveal that zoomorphic designs (e.g., the cat-like device) and even minimally anthropomorphic conversational interfaces (e.g., a voice assistant) can powerfully shape social expectations and engagement. By tracing these responses back to specific CASA cues show how particular cue configurations activate different relational expectations within our framework. Our results demonstrate how users interpret a device’s social role based on its behaviors, interactions, and perceived intent. These factors can be at least as influential as physical appearance or interactivity in shaping older adults’ responses and comfort levels. For instance, while a highly human like robot might elicit discomfort due to surveillance concerns when perceived as a ‘Warden,’ a subtly animated pet-like device can evoke genuine affection and companionship, showcasing the complex and non-linear nature of anthropomorphism’s impact.

Further, this study significantly contributes to the growing body of research on anthropomorphism by providing age specific insights, particularly from older adults. This demographic is often underrepresented or stereotyped in the IS field. While much existing research on human robot interaction has focused on younger or general adult populations (e.g., Benlian et al., 2020; Mourey et al., 2017; Touré-Tillery & McGill, 2015; Zheng & Jarvenpaa, 2021), this overlooks the unique life experiences, values, and socio-emotional needs that shape older adults’ engagement with novel technologies. Our framework meticulously categorizes how fundamental human needs, such as autonomy, the desire for companionship, and varying relational expectations, critically shape the acceptance and effectiveness of SARs within an aging population. We move beyond simplistic measures of acceptance (e.g., usefulness or ease of use) to illuminate how these deeper socio-emotional drivers map directly onto distinct device roles. For example, our work empirically validates conceptual ideas about older adults preferring ‘tools’ or ‘pets’ over ‘carers’ in some contexts, by providing concrete evidence of the characteristics and user responses associated with the Tool, Pet, Carer, and Warden roles. This systematic categorization and empirical grounding offer an unprecedentedly structured approach for designing SARs (Liu et al., 2023).

### Practical Contributions

This study offers actionable, empirically grounded guidelines for designing SARs that genuinely cater to older adults’ diverse and evolving needs. Our framework, encompassing Carer, Pet, Warden, and Tool roles, moves beyond purely functional designs, emphasizing the critical importance of aligning technology with older adults’ complex social and emotional needs to support both practical tasks and psychological wellbeing.

A key practical implication is the imperative for SARs to incorporate highly customizable and flexible anthropomorphic and zoomorphic features. This adaptability allows devices to respond to users’ emotional and practical needs, ultimately fostering autonomy and enhancing quality of life for aging individuals. After determining the most appropriate perceived role for a user, the SAR could then deliver the most suitable manifestation of that role. Users should control a device’s appearance, interaction style, and perceived social role to match their comfort level. For example, a SAR could offer a “Carer” mode with empathetic cues or a minimalist “Tool” mode, ensuring users retain control and allowing the device to shift roles as needed.

Our framework translates into specific design implications for each role:

- Tool Role: Designs aligned with this role should prioritize unobtrusive efficiency by minimizing anthropomorphic and behavioral cues. Physical cues should emphasize invisibility or seamless integration, such as embedding sensors into existing household objects, and behavioral cues should center on simple, user-initiated commands (e.g., hands free voice requests without unsolicited prompts).
- Warden Role: Devices targeting this role should adopt behavioral cues that support discreet, noninvasive monitoring while avoiding anthropomorphic signals that imply agency or intent. For example, fall detection could rely on passive infrared sensing rather than expressive or movement-based cues, paired with explicit opt-in controls that reinforce user autonomy.
- Carer Role: Devices oriented to this role must employ soft behavioral cues (e.g., gentle, optional check-ins) and moderate anthropomorphic cues that convey warmth without provoking overreach or surveillance concerns. Physical cues should remain subtle, enabling users to calibrate social presence through customizable interaction frequency and tone.
- Pet Role: Devices promoting this role benefit from warm physical cues (e.g., soft textures, lifelike movement) and moderate anthropomorphic cues that encourage emotional engagement without implying human-like oversight. Behavioral cues such as gentle haptic feedback, gaze-following, or subtle sound cues (e.g., purring) can support companionship while preserving user autonomy.

### Limitations

Despite some limitations, this study provides valuable insights into how older adults interact with anthropomorphized SARs. The relatively small, specific sample provides an in-depth exploration of older adults striving for autonomy, an increasingly relevant demographic. While this limits generalizability, these focused insights create a foundation for future research to expand upon by including a broader range of older adults, such as those in assisted living or with varying cognitive and physical abilities.

The controlled virtual environment ensured consistency in exploring user device interactions. While VR studies require caution when making generalizable claims (Holleman et al., 2020), this setting minimized external influences, providing a strong foundation for examining first impressions of anthropomorphized devices. Although real world settings may introduce additional variability, emerging research suggests VR findings often align with real world outcomes (Taufik et al., 2021). Future studies should extend this by examining actual interactions and long term behaviors in real world home environments, which would enhance ecological validity and provide deeper insight into sustained user engagement (Seeger et al., 2021). Additionally, while the vignette technique is valuable for eliciting immediate perceptions, subsequent research should further strengthen the generalizability of our framework by empirically validating it through quantitative or mixed methods approaches.

This study relies on self-reported data, gathered through interviews, and captures participants’ immediate, subjective responses. While self-reports can be influenced by social desirability bias and may not fully reflect long term perceptions, they offer valuable insight into first impressions, crucial for understanding early adoption patterns among older adults. Future longitudinal studies could complement these findings by tracking how relationships with SARs evolve over time. Finally, the study’s categorization of SARs into four distinct roles, provides a structured framework for understanding relational dynamics with anthropomorphized technology. While this framework may not capture every possible role as technology advances, it provides a basis for future research to refine and expand upon. As SARs become more sophisticated, this categorization can evolve to capture more complex interactions and emerging roles.

### Future Works

Building on the conceptual framework introduced in this study, future research can extend and deepen understanding of how older adults engage with SARs across diverse contexts. A first direction concerns broadening the participant base beyond independent community dwelling older adults. Including individuals in assisted living, those with cognitive or physical impairments, and participants from different cultural backgrounds would allow for richer cross-contextual insights. Such comparative research could illuminate how cultural values, caregiving traditions, and prior experiences with technology shape the meanings assigned to SARs and the acceptance of anthropomorphized forms.

A second direction involves exploring SAR roles within formal caregiving environments such as hospitals, rehabilitation centers, and residential care facilities. In these structured contexts, care routines, institutional norms, and professional boundaries may heavily influence how SARs are perceived, potentially amplifying or constraining certain relational roles. Investigating how caregivers and residents jointly construct the roles could reveal how institutional settings mediate autonomy, trust, and emotional comfort in human–robot relationships.

Third, there is considerable potential to examine the design implications of customizable anthropomorphism. Future studies might explore how adjustable features, such as appearance, tone of voice, or responsiveness, enable users to shape the social and emotional presence of SARs by selecting the cues they are most comfortable with. Personalization may allow technologies to better align with users’ relational preferences: for instance, some may prefer the warmth of a zoomorphic “Pet,” while others value the neutrality of a functional “Tool.” Understanding how customization impacts perceived autonomy, engagement, and resistance would provide crucial guidance for inclusive and adaptive SAR design.

Finally, longitudinal and ethnographic approaches are needed to examine how relationships with SARs evolve over time. Older adults’ perceptions are unlikely to remain static; familiarity, dependency, and life transitions (such as health deterioration) may all alter how technologies are interpreted and integrated into daily routines. Capturing these temporal dynamics could illuminate how social norms and emotional responses shift with sustained interaction, providing insight into the long-term relational trajectories of human–robot engagement.

Together, these research directions can move the field beyond questions of acceptance or usability toward a more nuanced understanding of how SARs become socially embedded over time. By examining diversity, context, personalization, and temporality, future work can further refine the relational framework developed here and inform the design of technologies that genuinely support older adults’ autonomy, dignity, and wellbeing.

## Concluding Remarks

This study offers an empirically grounded framework explaining how older adults perceive and engage with anthropomorphized SARs. By conceptualizing four relational roles, Carer, Pet, Warden, and Tool, it highlights how social interpretation and anthropomorphic embodiment jointly shape user experience. Rather than viewing acceptance as a linear process, the findings reveal that older adults actively negotiate the meaning of these technologies, aligning them with their own values of autonomy, safety, and emotional comfort.

The study contributes to broader debates on human–technology relationships by reframing adoption through a relational and interpretive lens. It demonstrates that older adults’ responses to SARs depend not only on functional performance but also on how these technologies are positioned within their social world, as companions, assistants, or instruments of control. In doing so, this research bridges theoretical perspectives from social robotics and ageing studies, offering a framework that future empirical work can test, refine, and extend.

## Data Availability

Data is not available to be shared, but analysis can be made available on request.

## Appendix B: Evidence in support of conceptual framework

**Table:**

	**Low Autonomy**	**High Autonomy**
High Relationship Tolerance	**Kitty** “A non-threatening physical entity like Kitty, just because I mean both forms become a habit when you get used to it.” (Arthur) “Think when you're thinking about robots, perhaps there's some resistance. But perhaps they're more acceptable. And this sounds ridiculous because you don't want a cat to take over, do you? But it's some sort of familiarity that you have when we have no experience of robots. But perhaps the cat draws on the experience you have of a home. Obviously, it's the same being a machine. But it sort of fits in better somehow.” (Clara) **Ziggy** “Yes, Ziggy could be nice. For example, if you're living on your own, you know, it might be reassuring to actually interact with something rather than the ether. Again, if it's... but you wouldn't react with a kitten or a puppy, would you? You'd want it to be a person. It comes easier from a robot rather than nothing in the house... because you’ve got a focus, yes, something that’s learning about you.” (Alice) “I'd be quite happy to sit and have a conversation with Ziggy about dressage and my memories of Portugal or whatever. Yeah, it'd be great.” (Olivia)	**Kitty** “But have something physically in the room with you. So, people might feel reassured by it, having an automatic kitten or puppy in the room. But then, if it was a kitten, you wouldn't be asking it questions. It needs to be a person.” (Alice) “This very quickly, in your imagination, could turn into Kitty, Kitty the cat, Pedro, or the puppy or whatever. And in that sense, I certainly remember talking to my puppies when I was younger. I'd sit on the stairs and tell them my life's history. I might not do that now, but I could see you could say something like, 'Hi Kitty, have I forgot my tablets again?' for it to turn around and say, 'Yes, you have. You've missed your 3 o'clocks.'” (David) “You feel, I think, more relaxed with it because we're used to animals, aren't you?” (Elizabeth) **Alexa** “I found it slightly creepy to have some little objects, looking like a cat, in there and being a presence in the home, where it's subtle. And it's imperceptible unless it's used. I'd be 100% happy with it.” (Joseph) “It is good, but I just prefer to talk. It's like as if I'm talking to something because I can see it.” (Henry) “No. Well, I think it's better being impartial and inanimate, in a sense. You cannot accuse someone of nagging, can you? Because it's what is basically data-driven. So, yeah, I think it's better than someone else telling you that.” (Leo)
Low Relationship Tolerance	**Ziggy** “I don't know. It's a child size. So it just seems very childlike, you know? You wouldn't expect to have a child looking after you.” (Hannah) “He moves in a much more ungainly way and much more mechanically. And that also seems better? Because he's making very nuanced little movements, which don't seem machine-like but just seem unusual to me.” (Sarah) “No, because he's too... he's much more human-like. I said, that's more scary.” (Sarah) **House** “I just think... I just don't like it. I just find it, I can't warm to it in any way.” (Arthur) “Because there are probably as many people on the opposite side of the fence to me who think, well, having a disembodied voice is like having ghosts in the house. So I can see it both ways, but my personal preference would not be something that's scooting up on me, behind me on the ankles all the time.” (Joseph)	**Alexa ** “No, I don't think so. I’d call a friend or something else, I think. Well, almost certainly, I wouldn't see that as a substitute. I find that a little bit weird in a way.” (Benjamin) “But if there are lots of electrical and electronic devices that are already part of your daily life, you don't have a relationship with them.” (Arthur) “There’s no companionship with an Alexa because it’s just a voice.” (Olivia) **House** “Strangely enough, the House one... my first thought was Star Trek, where, you know, you just speak and the answer is there. They didn't know how, but they got the concept, even all those years ago. So I would imagine anybody who watched Star Trek would have the seed sown that this could happen.” (Alice)

## Footnotes

1
https://www.alliancembs.manchester.ac.uk/about/our-campus/data-visualisation-observatory/.

## References

[CR1] Atzmüller, C., & Steiner, P. M. (2010). Experimental vignette studies in survey research. *Methodology*, *6*(3), 128–138. 10.1027/1614-2241/a000014

[CR2] Bartneck, C., Kulić, D., Croft, E., & Zoghbi, S. (2009). Measurement instruments for the anthropomorphism, animacy, likeability, perceived intelligence, and perceived safety of robots. *International Journal of Social Robotics*, *1*(1), 71–81. 10.1007/s12369-008-0001-3

[CR3] Barton, C., Sturge, G., & Harker, R. (2024). *The UK’S changing population*. House of Commons Library. https://commonslibrary.parliament.uk/the-uks-changing-population/#:~:text=An%20ageing%20population,but%20higher%20than%20some%20countries

[CR4] Benlian, A., Klumpe, J., & Hinz, O. (2020). Mitigating the intrusive effects of smart home assistants by using anthropomorphic design features: A multimethod investigation. *Information Systems Journal*, *30*(6), 1010–1042. 10.1111/isj.12243

[CR5] Bingley, W. J., Curtis, C., Lockey, S., Bialkowski, A., Gillespie, N., Haslam, S. A., Ko, R. K. L., Steffens, N., Wiles, J., & Worthy, P. (2023). Where is the human in human-centered AI? Insights from developer priorities and user experiences. *Computers in Human Behavior*. 10.1016/j.chb.2022.107617

[CR6] Bradwell, H. L., Edwards, K. J., Winnington, R., Thill, S., & Jones, R. B. (2019). Companion robots for older people: Importance of user-centred design demonstrated through observations and focus groups comparing preferences of older people and roboticists in South West England. *BMJ Open,**9*(9), Article e032468. 10.1136/bmjopen-2019-032468 10.1136/bmjopen-2019-032468 PMC6773331 31558461

[CR7] Braun, V., & Clarke, V. (2006). Using thematic analysis in psychology. *Qualitative Research in Psychology*, *3*(2), 77–101. 10.1191/1478088706qp063oa

[CR8] Choudrie, J., & Vyas, A. (2014). Silver surfers adopting and using facebook? A quantitative study of Hertfordshire, UK applied to organizational and social change. *Technological Forecasting and Social Change*, *89*, 293–305. 10.1016/j.techfore.2014.08.007

[CR9] Coeckelbergh, M. (2011). Humans, animals, and robots: A phenomenological approach to Human-Robot relations. *International Journal of Social Robotics,**3*(2), 197–204. 10.1007/s12369-010-0075-6

[CR10] Coghlan, S., Waycott, J., Lazar, A., & Barbosa Neves, B. (2021). Dignity, autonomy, and style of company: Dimensions older adults consider for robot companions. *Proceedings of the ACM on Human-Computer Interaction,**5*(CSCW1), 1–25. 10.1145/3449178 34308262 10.1145/3449178 PMC8297987

[CR11] De Carolis, B., Ferilli, S., & Palestra, G. (2017). Simulating empathic behavior in a social assistive robot. *Multimedia Tools and Applications,**76*(4), 5073–5094. 10.1007/s11042-016-3797-0

[CR12] De Graaf, M. M. A., & Allouch, S. B. (2017). The influence of prior expectations of a robot’s lifelikeness on users’ intentions to treat a zoomorphic robot as a companion. *International Journal of Social Robotics,**9*(1), 17–32. 10.1007/s12369-016-0340-4

[CR13] De Graaf, M. M. A., Allouch, S. B., & Klamer, T. (2015). Sharing a life with Harvey: Exploring the acceptance of and relationship-building with a social robot. *Computers in Human Behavior,**43*, 1–14. 10.1016/j.chb.2014.10.030

[CR14] De Graaf, M. M. A., Ben Allouch, S., & Van Dijk, J. A. G. M. (2019). Why would I use this in my home? A model of domestic social robot acceptance. *Human-Computer Interaction,**34*(2), 115–173. 10.1080/07370024.2017.1312406

[CR15] de Visser, E. J., Monfort, S. S., McKendrick, R., Smith, M. A. B., McKnight, P. E., Krueger, F., & Parasuraman, R. (2016). Almost human: Anthropomorphism increases trust resilience in cognitive agents. *Journal of Experimental Psychology: Applied,**22*(3), 331–349. 10.1037/xap0000092 27505048 10.1037/xap0000092

[CR16] Dickinson, P., Gerling, K., Wilson, L., & Parke, A. (2020). Virtual reality as a platform for research in gambling behaviour. *Computers in Human Behavior,**107*, Article 106293. 10.1016/j.chb.2020.106293

[CR17] Dosso, J. A., Kailley, J. N., Guerra, G. K., & Robillard, J. M. (2023). Older adult perspectives on emotion and stigma in social robots. *Frontiers in Psychiatry,**13*, Article 1051750. 10.3389/fpsyt.2022.1051750 36713914 10.3389/fpsyt.2022.1051750 PMC9878396

[CR18] Epley, N., Waytz, A., & Cacioppo, J. T. (2007). On seeing human: A three-factor theory of anthropomorphism. *Psychological Review,**114*(4), 864–886. 10.1037/0033-295X.114.4.864 17907867 10.1037/0033-295X.114.4.864

[CR19] Eyssel, F., & Hegel, F. (2012). (S)he’s got the look: Gender stereotyping of robots. *Journal of Applied Social Psychology*, *42*(9), 2213–2230. 10.1111/j.1559-1816.2012.00937.x

[CR20] Gambino, A., Fox, J., & Ratan, R. (2020). Building a stronger CASA: Extending the computers are social actors paradigm. *Human-Machine Communication*, *1*, 71–86. 10.30658/hmc.1.5

[CR21] Harrington, E. E., Bishop, A. J., Do, H. M., & Sheng, W. (2021). Perceptions of socially assistive robots: A pilot study exploring older adults’ concerns. *Current Psychology*. 10.1007/s12144-021-01627-5

[CR22] Heerink, M., Krose, B., Wielinga, B., & Evers, V. (2006). Human-Robot User Studies in Eldercare: Lessons Learned. In C. and A. Nugent (Ed.), *Smart Homes and Beyond* (Vol. 19, p. 31).

[CR23] Heerink, M., Kroese, B., Evers, V., & Wielinga, B. (2008). The Influence of Social Presence on Enjoyment and Intention to Use of a Robot and Screen Agent by Elderly Users. *RO-MAN 2008-The 17th IEEE International Symposium on Robot and Human Interactive Communication*, 695–700.

[CR24] Heerink, M., Krose, B., Evers, V., & Wielinga, B. (2010). Assessing acceptance of assistive social agent technology by older adults: The Almere model. *International Journal of Social Robotics*, *2*(4), 361–375. 10.1007/s12369-010-0068-5

[CR25] Hekkala, R., Stein, M., & Rossi, M. (2018). Metaphors in managerial and employee sensemaking in an information systems project. *Information Systems Journal*, *28*(1), 142–174. 10.1111/isj.12133

[CR26] Heyselaar, E. (2023). The CASA theory no longer applies to desktop computers. *Scientific Reports,**13*(1), Article 19693. 10.1038/s41598-023-46527-9 37952037 10.1038/s41598-023-46527-9 PMC10640629

[CR27] Holleman, G. A., Hooge, I. T. C., Kemner, C., & Hessels, R. S. (2020). The ‘Real-World approach’ and its problems: A critique of the term Ecological Validity. *Frontiers in Psychology,**11*, Article 721. 10.3389/fpsyg.2020.00721 32425850 10.3389/fpsyg.2020.00721 PMC7204431

[CR28] Ihamäki, P., & Heljakka, K. (2021). Robot pets as serious toys- Activating social and emotional experiences of elderly people. *Information Systems Frontiers*. 10.1007/s10796-021-10175-z 10.1007/s10796-021-10175-z PMC8364409 34413702

[CR29] Lee, L. Y., Lim, W. M., Teh, P. L., Malik, O. A. S., & Nurzaman, S. (2020). Understanding the interaction between older adults and soft service robots: Insights from robotics and the technology acceptance model. *AIS Transactions on Human-Computer Interaction*, 125–145. 10.17705/1thci.00132

[CR30] Li, J. (2015). The benefit of being physically present: A survey of experimental works comparing copresent robots, telepresent robots and virtual agents. *International Journal of Human-Computer Studies,**77*, 23–37. 10.1016/j.ijhcs.2015.01.001

[CR31] Liu, M., Wang, C., & Hu, J. (2023). Older adults’ intention to use voice assistants: Usability and emotional needs. *Heliyon,**9*(11), Article e21932. 10.1016/j.heliyon.2023.e21932 38027966 10.1016/j.heliyon.2023.e21932 PMC10663927

[CR32] Luger, E., & Sellen, A. (2016). ‘Like Having a Really Bad PA’: The Gulf between User Expectation and Experience of Conversational Agents. *Proceedings of the 2016 CHI Conference on Human Factors in Computing Systems*, 5286–5297. 10.1145/2858036.2858288.

[CR33] Łukasik, S., Tobis, S., Suwalska, J., Łojko, D., Napierała, M., Proch, M., Neumann-Podczaska, A., & Suwalska, A. (2021). The role of socially assistive robots in the care of older people: To assist in cognitive Training, to remind or to accompany? *Sustainability*, *13*(18), 10394. 10.3390/su131810394

[CR34] Ma, J. H., Erdogmus, E., Kangisser, S., & Yang, E. (2025). A comparative analysis of the effectiveness of immersive virtual reality on end-user design review. *Building and Environment,**267*, Article 112237. 10.1016/j.buildenv.2024.112237

[CR35] Marciano, A., & Nimrod, G. (2021). Identity collision: Older gay men using technology. *Journal of Computer-Mediated Communication*, *26*(1), 22–37. 10.1093/jcmc/zmaa016

[CR36] Mori, M., MacDorman, K. F., & Kageki, N. (2012). The uncanny Valley. *IEEE Robotics & Automation Magazine,**19*(2), 98–100. 10.1109/MRA.2012.2192811

[CR37] Mourey, J. A., Olson, J. G., & Yoon, C. (2017). Products as pals: Engaging with anthropomorphic products mitigates the effects of social exclusion. *Journal of Consumer Research,**44*(2), 414–431. 10.1093/jcr/ucx038

[CR38] Nass, C., & Moon, Y. (2000). Machines and mindlessness: Social responses to computers. *Journal of Social Issues*, *56*(1), 81–103. 10.1111/0022-4537.00153

[CR39] Nass, C., Steuer, J., & Tauber, E. R. (1994). Computers are Social Actors. *CHI 94 Celebrating Independence*, 72–78.

[CR40] Neo, J. R. J., Won, A. S., & Shepley, M. M. (2021). Designing immersive virtual environments for human behavior research. *Frontiers in Virtual Reality,**2*, Article 603750. 10.3389/frvir.2021.603750

[CR41] Nguyen, T. H., Waizenegger, L., & Techatassanasoontorn, A. A. (2022). Don’t neglect the user!” – Identifying types of human-chatbot interactions and their associated characteristics. *Information Systems Frontiers,**24*(3), 797–838. 10.1007/s10796-021-10212-x

[CR42] Niemelä-Nyrhinen, J. (2007). Baby boom consumers and technology: Shooting down stereotypes. *Journal of Consumer Marketing*, *24*(5), 305–312. 10.1108/07363760710773120

[CR43] Norouzi, N., Kim, K., Lee, M., Schubert, R., Erickson, A., Bailenson, J., Bruder, G., & Welch, G. (2019). Walking your virtual dog: Analysis of awareness and proxemics with simulated support animals in augmented reality. *2019 IEEE International Symposium on Mixed and Augmented Reality (ISMAR)*, *157*(168). 10.1109/ISMAR.2019.000-8

[CR44] Parsons, T. D. (2015). Virtual reality for enhanced ecological validity and experimental control in the clinical, affective and social neurosciences. *Frontiers in Human Neuroscience*. 10.3389/fnhum.2015.00660 10.3389/fnhum.2015.00660 PMC4675850 26696869

[CR45] Peek, S. T. M. M., Luijkx, K. G., Rijnaard, M. D., Nieboer, M. E., Van Der Voort, C. S., Aarts, S., van Hoof, J., Vrijhoef, H. J. M. M., & Wouters, E. J. M. M. (2016). Older adults’ reasons for using technology while aging in place. *Gerontology,**62*(2), 226–237. 10.1159/000430949 26044243 10.1159/000430949

[CR46] Pino, M., Boulay, M., Jouen, F., & Rigaud, A.-S. (2015). ‘Are we ready for robots that care for us?’ Attitudes and opinions of older adults toward socially assistive robots. *Frontiers in Aging Neuroscience,**7*, Article 141. 10.3389/fnagi.2015.00141 26257646 10.3389/fnagi.2015.00141 PMC4512026

[CR47] Portz, J. D., Bayliss, E. A., Bull, S., Boxer, R. S., Bekelman, D. B., Gleason, K., & Czaja, S. (2019). Using the technology acceptance model to explore user experience, intent to use, and use behavior of a patient portal among older adults with multiple chronic conditions: Descriptive qualitative study. *Journal of Medical Internet Research*. 10.2196/11604 10.2196/11604 PMC6475817 30958272

[CR48] Prakash, A., & Rogers, W. A. (2015). Why some humanoid faces are perceived more positively than others: Effects of Human-Likeness and task. *International Journal of Social Robotics*, *7*(2), 309–331. 10.1007/s12369-014-0269-4 26294936 10.1007/s12369-014-0269-4 PMC4539254

[CR49] Purington, A., Taft, J. G., Sannon, S., Bazarova, N. N., & Taylor, S. H. (2017). ‘Alexa is my new BFF’: Social Roles, User Satisfaction, and Personification of the Amazon Echo. *Proceedings of the 2017 CHI Conference Extended Abstracts on Human Factors in Computing Systems*, 2853–2859. 10.1145/3027063.3053246

[CR50] Robinson, H., MacDonald, B., & Broadbent, E. (2014). The role of healthcare robots for older people at home: A review. *International Journal of Social Robotics*, *6*(4), 575–591. 10.1007/s12369-014-0242-2

[CR51] Schneiders, E., Papachristos, E., & Van Berkel, N. (2021). The effect of embodied anthropomorphism of personal assistants on user perceptions. *Proceedings of the 33rd Australian Conference on Human-Computer Interaction*, *231-241*. 10.1145/3520495.3520503

[CR52] Schuetz, S., & Venkatesh, V. (2020). Research perspectives: The rise of human machines: How cognitive computing systems challenge assumptions of user-system interaction. *Journal of the Association for Information Systems*, *21*(2), 460–482. 10.17705/1jais.00608

[CR53] Seeger, A.-M., Pfeiffer, J., & Heinzl, A. (2021). Texting with humanlike conversational agents: Designing for anthropomorphism. *Journal of the Association for Information Systems,**22*(4), 931–967. 10.17705/1jais.00685

[CR54] Smarr, C.-A., Mitzner, T. L., Beer, J. M., Prakash, A., Chen, T. L., Kemp, C. C., & Rogers, W. A. (2014). Domestic robots for older adults: Attitudes, preferences, and potential. *International Journal of Social Robotics,**6*(2), 229–247. 10.1007/s12369-013-0220-0 25152779 10.1007/s12369-013-0220-0 PMC4138547

[CR55] Sommer, K. A., & Mabin, V. J. (2016). Insights into the eldercare conundrum through complementary lenses of Boardman’s SSM and TOC’s evaporating cloud. *European Journal of Operational Research,**248*(1), 286–300. 10.1016/j.ejor.2015.06.033

[CR56] Spaccatini, F., Corlito, G., & Sacchi, S. (2023). New dyads? The effect of social robots’ anthropomorphization on empathy towards human beings. *Computers in Human Behavior*. 10.1016/j.chb.2023.107821

[CR57] Storey, A. (2022). Voices of our ageing population: Living longer lives. In *Office for National Statistics*. https://www.ons.gov.uk/peoplepopulationandcommunity/birthsdeathsandmarriages/ageing/articles/voicesofourageingpopulation/livinglongerlives

[CR58] Sundar, S. S., Jung, E. H., Waddell, T. F., & Kim, K. J. (2017). Cheery companions or serious assistants? Role and demeanor congruity as predictors of robot attraction and use intentions among senior citizens. *International Journal of Human-Computer Studies,**97*, 88–97. 10.1016/j.ijhcs.2016.08.006

[CR59] Taufik, D., Kunz, M. C., & Onwezen, M. C. (2021). Changing consumer behaviour in virtual reality: A systematic literature review. *Computers in Human Behavior Reports,**3*, Article 100093. 10.1016/j.chbr.2021.100093

[CR60] Techatassanasoontorn, A. A., Waizenegger, L., & Doolin, B. (2023). When Harry, the human, met Sally, the software robot: Metaphorical sensemaking and sensegiving around an emergent digital technology. *Journal of Information Technology,**38*(4), 416–441. 10.1177/02683962231157426

[CR61] Touré-Tillery, M., & McGill, A. L. (2015). Who or what to believe: Trust and the differential persuasiveness of human and anthropomorphized messengers. *Journal of Marketing,**79*(4), 94–110. 10.1509/jm.12.0166

[CR62] Urquhart, C., Lehmann, H., & Myers, M. D. (2010). Putting the ‘theory’ back into grounded theory: Guidelines for grounded theory studies in information systems. *Information Systems Journal,**20*(4), 357–381. 10.1111/j.1365-2575.2009.00328.x

[CR63] Van Assche, M., Petrovic, M., Cambier, D., Calders, P., Van Gelder, P., & Van De Velde, D. (2024). The perspectives of older adults with mild cognitive impairment and their caregivers on the use of socially assistive robots in healthcare: Exploring factors that influence attitude in a pre-implementation stage. *Disability and Rehabilitation: Assistive Technology,**19*(1), 222–232. 10.1080/17483107.2022.2075477 35587020 10.1080/17483107.2022.2075477

[CR64] Vandemeulebroucke, T., Dierckx De Casterlé, B., & Gastmans, C. (2020). Ethics of socially assistive robots in aged-care settings: A socio-historical contextualisation. *Journal of Medical Ethics,**46*(2), 128–136. 10.1136/medethics-2019-105615 31818967 10.1136/medethics-2019-105615

[CR65] Whelan, S., Murphy, K., Barrett, E., Krusche, C., Santorelli, A., & Casey, D. (2018). Factors affecting the acceptability of social robots by older adults including people with dementia or cognitive impairment: A literature review. *International Journal of Social Robotics*, *10*(5), 643–668. 10.1007/s12369-018-0471-x

[CR66] Wu, H., Cai, T., Luo, D., Liu, Y., & Zhang, Z. (2021). Immersive virtual reality news: A study of user experience and media effects. *International Journal of Human-Computer Studies,**147*, Article 102576. 10.1016/j.ijhcs.2020.102576

[CR67] Zheng, J. F., & Jarvenpaa, S. L. (2021). Thinking technology as human: Affordances, technology features, and egocentric biases in technology anthropomorphism. *Journal of the Association for Information Systems,**22*(5), 1429–1453. 10.17705/1jais.00698

[CR68] Zsiga, K., Edelmayer, G., Rumeau, P., Peter, O., Toth, A., & Fazekas, G. (2013). Home care robot for socially supporting the elderly: Focus group studies in three European countries to screen user attitudes and requirements. *International Journal of Rehabilitation Research*, *36*(4), 375–378. 10.1097/MRR.0b013e3283643d26 24189106 10.1097/MRR.0b013e3283643d26

